# Effect of community health clubs on child diarrhoea in western Rwanda: cluster-randomised controlled trial

**DOI:** 10.1016/S2214-109X(17)30217-6

**Published:** 2017-06-12

**Authors:** Sheela S Sinharoy, Wolf-Peter Schmidt, Ronald Wendt, Leodomir Mfura, Erin Crossett, Karen A. Grépin, William Jack, Bernard Ngabo Rwabufigiri, James Habyarimana, Thomas Clasen

**Affiliations:** a Department of Environmental Health, Rollins School of Public Health, Emory University, Atlanta, GA, USA; b Faculty of Infectious and Tropical Diseases, London School of Hygiene and Tropical Medicine, London, UK; c Innovations for Poverty Action, New Haven, CT, USA; d McCourt School of Public Policy, Georgetown University, Washington, DC, USA; e Department of Economics, Georgetown University, Washington, DC, USA; f Department of Health Sciences, Wilfrid Laurier University, Waterloo, ON, Canada; g School of Public Health, National University of Rwanda, Kigali, Rwanda

## Abstract

**Background:**

Community health clubs are multi-session village-level gatherings led by trained facilitators and designed to promote healthy behaviours mainly related to water, sanitation, and hygiene. They have been implemented in several African and Asian countries but have never been evaluated rigorously. We aimed to evaluate the effect of two versions of the community health club model on child health and nutrition outcomes.

**Methods:**

We did a cluster-randomised trial in Rusizi district, western Rwanda. We defined villages as clusters. We assessed villages for eligibility then randomly selected 150 for the study using a simple random sampling routine in Stata. We stratified villages by wealth index and by the proportion of children younger than 2 years with caregiver-reported diarrhoea within the past 7 days. We randomly allocated these villages to three study groups: no intervention (control; n=50), eight community health club sessions (Lite intervention; n=50), or 20 community health club sessions (Classic intervention; n=50). Households in these villages were enrolled in 2013 for a baseline survey, then re-enrolled in 2015 for an endline survey. The primary outcome was caregiver-reported diarrhoea within the previous 7 days in children younger than 5 years. Analysis was by intention to treat and per protocol. This trial is registered with ClinicalTrials.gov, number NCT01836731.

**Findings:**

At the baseline survey undertaken between May, 2013, and August, 2013, 8734 households with children younger than 5 years of age were enrolled. At the endline survey undertaken between Sept 21, 2015, and Dec 22, 2015, 7934 (91%) of the households were re-enrolled. Among children younger than 5 years, the prevalence of caregiver-reported diarrhoea in the previous 7 days was 514 (14%) of 3616 assigned the control, 453 (14%) of 3196 allocated the Lite intervention (prevalence ratio compared with control 0·97, 95% CI 0·81–1·16; p=0·74), and 495 (14%) of 3464 assigned the Classic intervention (prevalence ratio compared with control 0·99, 0·85–1·15; p=0·87).

**Interpretation:**

Community health clubs, in this setting in western Rwanda, had no effect on caregiver-reported diarrhoea among children younger than 5 years. Our results question the value of implementing this intervention at scale for the aim of achieving health gains.

**Funding:**

Bill & Melinda Gates Foundation.

## Introduction

The importance of good health and nutrition for children younger than 5 years is widely recognised. The recently adopted Sustainable Development Goals include goals such as zero hunger (Goal 2), good health and wellbeing (Goal 3), and clean water and sanitation (Goal 6), which reflect the global community's prioritisation of the need to improve food and nutrition security as well as coverage of improved water and sanitation.^1^

The Government of Rwanda has made a commitment to improving the health and nutrition of its children.^2^, ^3^ The 2014–15 Demographic and Health Survey documents a steady decline in the proportion of children who are chronically undernourished (stunted), from 51% in 2005 to 38% in 2014–15.^4^ However, in these data, the prevalence of caregiver-reported diarrhoea has declined only slightly, from 14% in 2005 to 12% in 2014–15, possibly attributable in part to deficiencies in water quality, sanitation, and hygiene (WASH) practices.^4^

As part of a strategy to address the continued high prevalence of diarrhoea, the Rwandan Ministry of Health launched the Community-Based Environmental Health Promotion Programme (CBEHPP).^5^ CBEHPP used the community health club approach to promote healthy practices, with the aim of achieving zero open defecation, at least 80% hygienic latrine coverage, and improvements in related health behaviours such as household water treatment and handwashing with soap.^5^ Similar group-based approaches at the community level in sub-Saharan Africa have been shown to have positive effects on infant mortality and other health and nutrition outcomes, but few studies have shown an effect on behaviours relating to WASH.^6^, ^7^ The objective of our study was to evaluate the effect of the CBEHPP model on child diarrhoea, child anthropometry, and household water quality.

**Research in context**

**Evidence before this study**

Before this study, systematic reviews had shown that interventions designed to improve water quality, sanitation, and hygiene (WASH) practices were generally effective at preventing reported diarrhoea among young children in low-income settings. There was less evidence that WASH interventions could improve children's nutritional status. In general, reviews found the underlying evidence to be of low quality. Moreover, randomised controlled trials of WASH interventions delivered programmatically showed no protective effect against diarrhoea, possibly because of poor delivery and uptake of the intervention. Community health clubs had been promoted in many countries in Africa and southeast Asia as a means of improving WASH practices and nutrition. However, they had never been evaluated rigorously for their effect on health or other outcomes.

**Added value of this study**

The results of our cluster-randomised controlled trial show no effect of a community health club intervention on caregiver-reported diarrhoea among children younger than 5 years or on nutritional status among infants younger than 2 years. We noted positive effects of community health clubs on some household-level intermediate outcomes within villages allocated the full-length intervention (ie, 20 community health club sessions), but these did not translate into improvements in individual-level health and nutrition outcomes. Approaches that use group education sessions for behaviour change with the aim of improving maternal and child health and nutrition are common in low-income and middle-income countries. Our study suggests that these approaches might not be effective in producing measurable improvements in health and nutrition.

**Implications of all the available evidence**

There is a need for more rigorous investigations to identify low-cost interventions that are effective at improving child health and nutrition, either independently or in combination. These studies will need to assess programme delivery and adherence to understand whether provision of interventions can be improved to achieve greater effects.

## Methods

### Study design

We did a cluster-randomised controlled trial to assess the CBEHPP and community health club approach in a district of Rwanda. To choose a location for the study, we worked with the Rwandan Ministry of Health and the programme implementer to select a district based on two criteria. First, the district had to have no previous implementation of CBEHPP and no existing donor commitment to support implementation. Four of 30 districts nationwide met this first criterion. Second, we sought a district with a high burden of the diseases targeted by the planned intervention. Using existing administrative data and focusing on diseases such as malaria and helminth infection, the implementation and research team examined the composition and severity of the disease burden. The disease burden and absence of donors across these four districts are strong predictors for a high poverty concentration. We selected Rusizi district of western Rwanda for this trial.

In Rwanda, districts are divided into sectors, followed by cells, then villages. Cells contain two to eight villages. Villages, as defined by the National Institute of Statistics Rwanda (NISR), are the smallest geographical unit to describe the allocation of the population. Village size is heterogeneous: data from the national census of 2012 indicate that the mean village population is 600 (SD 300). For our trial, we defined a cluster as a village. The intervention entailed formation of community health clubs at the village level.

The protocol for this study was reviewed and approved by the Rwandan National Ethics Committee and the Institutional Review Board of Innovations for Poverty Action. We obtained written informed consent from the main respondent before interview.

### Randomisation and masking

Of 598 villages in Rusizi district, we randomly selected 150 to take part in our trial (figure). We did the random selection of villages in collaboration with NISR, which shared a recently updated sampling frame of sectors, cells, and villages for Rusizi district but did not share any geographical identifiers. To minimise contamination, we randomly selected five complete study samples of 150 villages each, then requested that NISR map the five samples. Based on the resulting maps, we chose the one sample that minimised the number of villages with shared borders. For each of the five samples, the study team had used a simple random sampling routine in Stata to select at most two villages from large cells (≥3 villages) and one village from small cells (<3 villages). We then randomly sorted the cells and selected the villages numbered from 1 to 150 to comprise the study sample.

**Figure:**
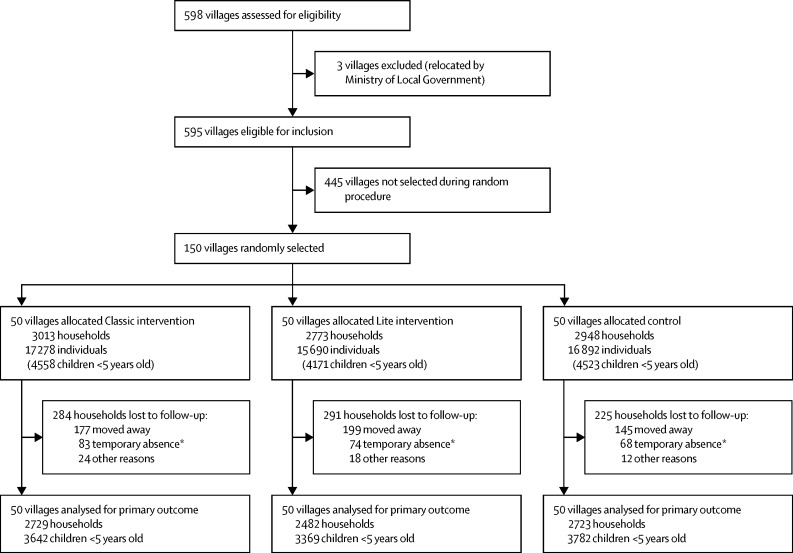
Trial profile No villages were lost to follow-up. *Temporary absence defined as being unavailable after data collectors visited the household three times in 1 day, with an interval of at least 2 h between each visit.

We did a baseline survey of the 150 villages, which included water sampling.^8^ To increase the study's power to accurately detect improvements in child health, we stratified the 150 villages along two dimensions: average fraction of children younger than 2 years with caregiver-reported diarrhoea in the previous 7 days; and average wealth index, which is a standardised variable (mean 0·04 [SD 0·67]). The wealth index is a weighted average of a household's ownership of 17 assets in three categories: durable goods such as refrigerator, television, and bicycle; large livestock such as cattle, goats, or sheep; and attributes of the housing structure, such as the roofing, wall, and floor materials. Data for both diarrhoea and wealth came from the baseline survey. Along both of these dimensions, we divided the sample into three equally sized groups representing high, moderate, and low levels of diarrhoea and wealth. We then cross-tabulated these in a 3 × 3 table that had a total of nine cells. Within each cell, we used Stata to randomly order the villages and divide them into three groups with approximately the same number of villages in each group. The first group of villages in each cell was allocated to the control arm of the study, the second to a “Lite” (shortened-duration) intervention arm, and the third to a “Classic” (full-length) intervention arm. This produced three study groups with 50 villages in each group. In this way, we aimed to ensure study balance along wealth, which, as a predictor of our main outcomes, could otherwise undermine inference.^9^, ^10^

One of us (JH) randomly allocated the 150 villages into one of the three study groups. The evaluation team, including data collectors, played no part in implementation of the intervention. The leadership of the community health club in each village managed the enrolment of participants into health clubs. To our knowledge, enrolment and participation in community health clubs was voluntary. Because of the nature of the intervention, masking was not possible at the participant or implementer level. It was also not possible to mask treatment status during data collection because of the nature of the survey questions, which pertained to participation in the health clubs.

### Procedures

Full details of the intervention background and activities are described elsewhere.^8^ Briefly, community health clubs in villages allocated to the Lite intervention held eight sessions on village mapping, personal hygiene, handwashing, diarrhoea, water sources, safe storage of drinking water, treatment of drinking water, and sanitation. The Classic intervention included 20 sessions, consisting of all the Lite sessions plus common diseases, skin diseases, infant care (weaning and immunisation), worms and intestinal parasites, food hygiene, nutrition, food safety and food security, the model home, good parenting, respiratory disease, malaria, bilharzia (schistosomiasis), and HIV/AIDS. In each village, the facilitator determined the meeting times and frequency. All sessions were open to any community members, with no inclusion or exclusion criteria. Most sessions had associated homework assignments to reinforce participants' learning. Implementation of the community health club model was consistent with the approach described elsewhere.^11^

For both interventions, community health club sessions were typically led by the In-Charge of Social Affairs (referred to in this report as the facilitator), who is one of five members of the village-level Executive Committee. Facilitators in villages assigned to the Classic intervention completed 5 days of training in February, 2014, and facilitators in villages allocated the Lite intervention had 2 days of training in June, 2014. The facilitators were free to start organising community health club sessions in their villages at any point after completion of the training. The starting dates and frequency of meetings varied widely across villages, depending on the facilitator and on other events such as village meetings, weddings, funerals, and rain. Facilitators in villages assigned the Classic intervention, but not those allocated the Lite intervention, had a training manual and visual aids. Community health clubs in villages allocated the Classic intervention also had attendance cards and organised graduation ceremonies, at which participants received certificates. We offered no other material incentives for participation. Implementation of community health clubs was complete in all intervention villages by March, 2015.

In the baseline survey, we targeted for inclusion all households with a child younger than 5 years in the study area, with no exclusion criteria. Data collection methods for the baseline survey have been described elsewhere.^8^ Methods for the endline survey followed those of the baseline, including a structured survey, observation, and collection of drinking water samples from roughly 10% of households. All follow-up, including water sampling, took place during one visit. For the endline survey, data collectors attempted to visit all households that had enrolled in the baseline survey. If the data collector visited a household three times in 1 day, with an interval of at least 2 h between each visit, and was not able to enrol the household, we recorded the household as a temporary absence. In the endline survey, we gathered data for caregiver-reported diarrhoea prevalence and anthropometric measurements for all children younger than 5 years. The baseline survey included length measurements for children younger than 2 years and weight measurements for children younger than 5 years; therefore, some older children in the endline survey had data from both baseline and endline surveys.

All data collectors were trained in pairs to obtain anthropometric measurements according to a standard procedure. The training included standardisation sessions at local health centres.^12^, ^13^ We retrained any pairs of data collectors whose interobserver reliability was outside a predetermined range, and the pair attended another standardisation session to confirm improvement.

We weighed all children younger than 5 years once, to the nearest 20 g (for weight <20 kg) or 50 g (for weight between 20 kg and 50 kg) using a Seca 385 scale (Seca, Hamburg, Germany). We measured length (for children <24 months) to the nearest 0·1 cm with Seca 417 length boards and height (for children ≥24 months) to the nearest 0·1 cm with Seca 213 stadiometers. We measured length and height in duplicate; we calculated the average of the two measurements for the analysis. If the two measurements differed by more than 0·7 cm, we asked data collectors to obtain a third length or height measurement on the spot, and we averaged the two closest measurements.

We obtained data for demographics (eg, child age and sex) and validated children's birth dates through immunisation cards when possible. We calculated stunting (defined as height-for-age or length-for-age *Z* score <–2) and wasting (defined as weight-for-height or weight-for-length *Z* score <–2) for descriptive purposes.

We collected data for attendance at community health club sessions through self-report, by which respondents were asked to recall which sessions, if any, they or anyone else in their household had attended. They were then prompted by data collectors, who read aloud a list of sessions and asked whether the respondent or anyone else in the household had attended each session. We combined the self-reported and prompted responses into one continuous variable for number of sessions attended by the household. The main treatment variable was the intervention status of the village where the individual lived at baseline.

### Outcomes

The primary outcome was caregiver-reported diarrhoea among children younger than 5 years in the past 7 days, at endline (roughly 2 years after baseline). We used the WHO definition of diarrhoea, which is three or more loose stools (that can take the shape of a container) within a 24-h period.^14^ Secondary outcomes were height-for-age or length-for-age *Z* score and weight-for-height or weight-for-length Z score for children younger than 5 years who were measured at baseline. We calculated these *Z* scores using WHO growth standards.^15^ An additional secondary outcome was colony-forming units of thermotolerant (faecal) coliforms per 100 mL water, which we calculated as described elsewhere.^8^

We also gathered data for intermediate outcomes, including improved drinking water source; household water treatment (boiling, filtration, chlorination, or solar disinfection); presence of improved sanitation facility; and sanitary disposal of children's faeces. We measured all these outcomes using a standard module from WHO and UNICEF.^16^ Data collectors also recorded the structure of sanitation facility (presence of floors, walls, and a roof); presence of faeces (human, animal, or both) in the household courtyard; and presence of a handwashing station with soap and water.

Other intermediate outcomes related to nutrition and food security. These included exclusive breastfeeding for infants younger than 6 months and dietary diversity for children aged 6–23 months, measured using a standard module from WHO.^17^ We measured household food security using the standard household hunger scale module from Food and Nutrition Technical Assistance (FANTA) III.^18^ Nutrition and food security variables rely on self-report by the main respondent or primary caregiver.

In addition to outcomes reported here, we gathered data for secondary endpoints including clinical data for diarrhoea and malaria and data for infant and child mortality. Those outcomes will be reported elsewhere.

### Statistical analysis

To calculate the sample size for our primary outcome variable (caregiver-reported diarrhoea in children aged <5 years in the past 7 days), we made several assumptions—namely, 85 children per village, a diarrhoea prevalence of 8% in villages allocated no intervention (control), a 25% reduction in diarrhoea attributable to the intervention, and an intravillage correlation of 0·0067. These data were informed by findings of previous diarrhoea studies and were updated based on baseline data.^8^, ^19^ Using a standard formula for the comparison of two proportions, and a design effect calculated on the basis of the intravillage correlation and village size, we estimated that 50 villages per arm would yield 80% power to detect the 25% reduction in diarrhoea.

We calculated descriptive statistics for each study group at baseline. Analysis of primary, secondary, and intermediate outcomes was by intention to treat and per protocol (for the Classic intervention only) at household and individual levels. For dichotomous outcomes at the individual level, we used log-binomial regression with a log-link function and generalised estimating equations (GEE) to account for village-level clustering, then calculated the exponential of the coefficients to obtain prevalence ratios (PRs). For dichotomous outcomes at the household level, we used binomial regression with an identity link function and GEE to obtain risk differences (RDs). For the ordinal variable representing household food security, we used ordinal logistic regression then calculated the exponential of the coefficients to obtain odds ratios (ORs). For continuous variables, we used linear regression with GEE to account for village-level clustering.

The primary analysis of anthropometric data included children measured both at baseline and endline. These children were all younger than 2 years at the time of the baseline survey and, hence, were aged 2–4 years at the time of the endline survey. Additional analyses included all children with anthropometric data, including but not restricted to those with baseline anthropometric data. For the main analysis of the effect of the interventions on height-for-age *Z* score and weight-for-height *Z* score among children who had been measured at baseline, we included that child's baseline length-for-age *Z* score or weight-for-length *Z* score, respectively. The adjustment for baseline values was done to improve precision of the estimates. The anthropometric analysis including all children was further stratified for children younger than 2 years and infants younger than 1 year, because the first two years of life are judged an important window for child growth.^20^, ^21^ No baseline adjustment was possible for these calculations.

In all analyses, we estimated coefficients for villages assigned the Classic and Lite interventions as differences between these villages relative to those allocated the control. We did not adjust for imbalance between study groups at baseline, because the size of the differences was small.

In the per-protocol analysis, we defined compliance as self-reported attendance of any household members at all 20 sessions. To investigate a potential dose-response relation between session attendance and effect size, we also calculated the effect size in those attending between one and 20 sessions. Because the per-protocol analysis showed no evidence for an effect, we did not further subdivide by the number of sessions. To reduce the risk of bias in the per-protocol analysis, we adjusted for baseline values of each outcome variable.

All analyses were done using Stata version 14.1. This trial is registered with ClinicalTrials.gov, number NCT01836731.

### Role of the funding source

The funder had no role in study design, data collection, data analysis, data interpretation, or writing of the report. The corresponding author had full access to all the data in the study and had final responsibility for the decision to submit for publication.

## Results

Between May, 2013, and August, 2013, the baseline survey of households was done, and between Sept 21, 2015, and Dec 22, 2015, households were re-enrolled for the endline survey. Of 8734 households that consented to participate in the baseline survey, 7934 (91%) households gave written consent to participate in the endline survey, with no difference in attrition between intervention groups (figure). The most common reasons for loss to follow-up were because the household moved away or because of a temporary absence, defined as being unavailable after data collectors visited the household three times in 1 day, with an interval of at least 2 h between each visit. Of households that moved between the baseline and endline surveys, most moved outside the study area. Six households moved within the study area and were enrolled in the endline survey. Only one of these households moved to a village allocated a different intervention; this household was included in analyses based on its location at baseline. At baseline, anthropometric measurements were obtained for 4598 children younger than 2 years; 3872 older children in the endline survey had anthropometric data from both baseline and endline surveys, whereas 5606 children had only post-intervention anthropometric data.

Table 1 shows selected characteristics of children and households in the study at baseline, by study group. Data for 13 252 children younger than 5 years were obtained at baseline. No differences were recorded between intervention groups for the main outcomes of diarrhoea, mean length-for age *Z* score, mean weight-for-length *Z* score, stunting, wasting, or mean thermotolerant coliforms per 100 mL water. The villages assigned the control had a slightly lower prevalence of sanitation facilities with complete floor, walls, and roof, and a lower prevalence of no visible faeces in the courtyard. Villages allocated the Lite intervention had a slightly lower prevalence of improved sources of drinking water. Exclusive breastfeeding, dietary diversity, and household food security were not measured at baseline and are therefore not included in table 1.

**Table 1:** Baseline characteristics

		**Control**	**Lite**	**Classic**
**Children younger than 5 years**				
Diarrhoea in previous 7 days		375/4307 (9%)	349/3954 (9%)	380/4312 (9%)
Stunted		557/1615 (34%)	495/1421 (35%)	534/1550 (34%)
	Height-for-age *Z* score	−1·47 (1·41)	−1·53 (1·36)	−1·49 (1·43)
Wasted		31/1619 (2%)	35/1422 (2%)	31/1557 (2%)
	Weight-for-height *Z* score	0·28 (1·12)	0·23 (1·14)	0·30 (1·14)
Male sex		2167/4307 (50%)	2020/3954 (51%)	2122/4312 (49%)
Female sex		2140/4307 (50%)	1934/3954 (49%)	2190/4312 (51%)
Age (months)				
	<12	847/4307 (20%)	716/3954 (18%)	796/4312 (18%)
	12–23	883/4307 (20%)	816/3954 (21%)	865/4312 (20%)
	24–59	2577/4307 (60%)	2423/3954 (61%)	2651/4312 (61%)
Duration of maternal education (years)		4·2 (3·1)	4·2 (3·0)	4·1 (3·0)
**Household level**				
Thermotolerant coliforms per 100 mL water (colony-forming units)		126·1 (216·7)	136·2 (230·1)	156·9 (258·1)
	Number of households*	426	431	448
Drinking water obtained from an improved source†		2241/2948 (76%)	2008/2760 (73%)	2275/2989 (76%)
Treatment of drinking water is adequate		926/2911 (32%)	862/2733 (32%)	943/2939 (32%)
Improved sanitation facility‡		1952/2948 (66%)	1868/2760 (68%)	2030/2989 (68%)
Structurally complete sanitation facility (ie, floor, walls, and roof)		148/2911 (5%)	182/2733 (7%)	196/2939 (7%)
Faeces visible in courtyard		449/2948 (15%)	355/2760 (13%)	388/2988 (13%)
Handwashing station observed, with soap and water		47/2948 (2%)	28/2760 (1%)	29/2988 (1%)
Sanitary disposal of child faeces (for children <3 years)		2665/2948 (90%)	2533/2760 (92%)	2692/2989 (90%)
Wealth quintile				
	First	599/2935 (20%)	562/2745 (20%)	602/2998 (20%)
	Second	620/2935 (21%)	546/2745 (20%)	575/2998 (19%)
	Third	613/2935 (21%)	544/2745 (20%)	661/2998 (22%)
	Fourth	563/2935 (20%)	576/2745 (21%)	548/2998 (18%)
	Fifth	540/2935 (18%)	517/2745 (19%)	612/2998 (20%)

Data are number of children younger than 5 years or number of households (%), or mean (SD).

* Water sampling was done in 10% of all study households.

† As defined by WHO and UNICEF, improved drinking water sources include piped water, public taps, tubewells, protected dug wells or springs, and rainwater.

‡ As defined by WHO and UNICEF, improved sanitation includes flush toilet, ventilated improved pit latrine, pit latrine with slab, and composting toilet.

Table 2 shows characteristics of households and children at endline. Data for 10 793 children younger than 5 years were obtained at endline. Among children younger than 5 years, the prevalence of caregiver-reported diarrhoea in the previous 7 days was 514 (14%) of 3616 assigned the control, 453 (14%) of 3196 allocated the Lite intervention, and 495 (14%) of 3464 assigned the Classic intervention. Among children younger than 2 years, the mean length-for-age *Z* score was −1·54 (SD 1·25) for those allocated control, −1·59 (1·28) for those assigned the Lite intervention, and −1·61 (1·32) for those assigned the Classic intervention, and mean weight-for-length *Z* scores were, respectively, 0·18 (SD 1·11), 0·18 (1·09), and 0·10 (1·11). The mean level of thermotolerant coliforms per 100 mL water was 139·8 colony-forming units (SD 230·9) in villages assigned the control, 164·1 (250·4) in villages allocated the Lite intervention, and 156·2 (244·1) in villages assigned the Classic intervention.

**Table 2:** Endline characteristics

		**Control**	**Lite**	**Classic**
**Children measured at baseline and endline**				
Height-for-age *Z* score		−1·89 (1·06)	−1·85 (1·10)	−1·84 (1·09)
	Number of children	1378	1183	1307
Weight-for-height *Z* score		0·067 (0·92)	0·032 (0·92)	0·085 (0·95)
	Number of children	1383	1180	1309
**Children younger than 5 years**				
Diarrhoea		514/3616 (14%)	453/3196 (14%)	495/3464 (14%)
Height-for-age *Z* score		−1·74 (1·18)	−1·78 (1·20)	−1·75 (1·23)
	Number of children	3320	2964	3190
Weight-for-height *Z* score		0·077 (0·98)	0·075 (0·98)	0·051 (1·00)
	Number of children	3284	2929	3134
Stunted		1410/3320 (42%)	1264/2964 (43%)	1303/3190 (41%)
Wasted		51/3284 (2%)	47/2929 (2%)	67/3134 (2%)
**Children younger than 2 years**				
Diarrhoea		232/1210 (19%)	234/1101 (21%)	249/1181 (21%)
Length-for-age *Z* score		−1·54 (1·25)	−1·59 (1·28)	−1·61 (1·32)
	Number of children	1095	1002	1081
Weight-for-length *Z* score		0·18 (1·11)	0·18 (1·09)	0·10 (1·11)
	Number of children	1065	976	1032
Stunted		405/1095 (37%)	375/1002 (37%)	404/1081 (37%)
Wasted		23/1065 (2%)	24/976 (2%)	25/1032 (2%)
**Children younger than 1 year**				
Diarrhoea		109/644 (17%)	109/547 (20%)	102/575 (18%)
Length-for-age *Z* score		−1·21 (1·29)	−1·14 (1·21)	−1·22 (1·34)
	Number of children	602	511	533
Weight-for-length *Z* score		0·31 (1·17)	0·27 (1·12)	0·17 (1·18)
	Number of children	598	512	532
Stunted		163/602 (27%)	119/511 (23%)	125/533 (23·5%)
Wasted		14/598 (2%)	15/512 (3%)	15/532 (3%)
**Household level**				
Thermotolerant coliforms per 100 mL water (colony-forming units)		139·8 (230·9)	164·1 (250·4)	156·2 (244·1)
	Number of households*	362	341	379
Drinking water obtained from an improved source†		2134/2723 (78%)	1814/2471 (73%)	2225/2721 (82%)
Treatment of drinking water is adequate		1101/2720 (41%)	1121/2466 (45%)	1326/2720 (49%)
Improved sanitation facility‡		805/2723 (30%)	733/2471 (30%)	1009/2721 (37%)
Structurally complete sanitation facility (ie, floor, walls, and roof)		695/2638 (26%)	620/2414 (26%)	849/2620 (32%)
Faeces visible in courtyard		249/2723 (9%)	265/2473 (11%)	245/2720 (9%)
Handwashing station observed, with soap and water		47/2723 (2%)	26/2470 (1%)	42/2721 (2%)
Sanitary disposal of child faeces (for children <3 years)		1115/1818 (61%)	983/1585 (62%)	1045/1737 (60%)
Household hunger (food security)				
	Little to none	1476/2723 (54%)	1348/2470 (55%)	1379/2721 (51%)
	Moderate	1020/2723 (37%)	966/2470 (39%)	1096/2721 (40%)
	Severe	227/2723 (8%)	156/2470 (6%)	246/2721 (9%)
**Child level**				
Exclusive breastfeeding (for infants <6 months)		241/311 (77%)	218/283 (77%)	231/302 (76%)
Minimum dietary diversity (for children aged 6–23 months·)		337/930 (36%)	320/844 (38%)	353/909 (39%)

Data are number of children or number of households (%), or mean (SD).

* Water sampling was done in 10% of all study households.

† As defined by WHO and UNICEF, improved drinking water sources include piped water, public taps, tubewells, protected dug wells or springs, and rainwater.

‡ As defined by WHO and UNICEF, improved sanitation includes flush toilet, ventilated improved pit latrine, pit latrine with slab, and composting toilet.

Table 3 shows the effect of the interventions on primary, secondary, and intermediate outcomes at the individual and household level (analysed by intention to treat). Neither the Lite intervention nor the Classic intervention had any effect on diarrhoea, height-for-age or length-for-age *Z* scores, or weight-for-height or weight-for-length *Z* scores among children who had been measured at baseline or those younger than 5 years, younger than 2 years, or younger than 1 year at endline. The Lite and Classic interventions also had no effect on water quality, as measured by thermotolerant coliforms per 100 mL water.

**Table 3:** Intention-to-treat analysis of primary, secondary, and intermediate outcomes

	n*	**Lite *vs* Control**		**Classic *vs* Control**	
		Estimate (95% CI)	p	Estimate (95% CI)	p
**Children measured at baseline and endline**					
Height-for-age *Z* score†	3869	0·054 (−0·065 to 0·17)	0·38	0·028 (−0·083 to 0·14)	0·62
Weight-for-height *Z* score‡	3874	−0·0024 (−0·079 to 0·074)	0·95	0·015 (−0·058 to 0·088)	0·69
**Children younger than 5 years**					
Diarrhoea (primary outcome)	10 276	0·97 (0·81 to 1·16)	0·74	0·99 (0·85 to 1·15)	0·87
Height-for-age *Z* score	9473	−0·0048 (−0·16 to 0·15)	0·95	−0·019 (−0·16 to 0·12)	0·79
Weight-for-height *Z* score	9346	−0·016 (−0·095 to 0·062)	0·68	−0·013 (−0·091 to 0·065)	0·75
**Children younger than 2 years**					
Diarrhoea	3492	1·07 (0·86 to 1·32)	0·57	1·08 (0·89 to 1·32)	0·42
Length-for-age *Z* score	3178	−0·036 (−0·18 to 0·11)	0·63	−0·077 (−0·23 to 0·075)	0·32
Weight-for-length *Z* score	3073	−0·0096 (−0·12 to 0·10)	0·87	−0·069 (−0·18 to 0·045)	0·23
**Children younger than 1 year**					
Diarrhoea	1766	1·16 (0·88 to 1·52)	0·30	1·03 (0·77 to 1·38)	0·84
Length-for-age *Z* score	1646	0·058 (−0·13 to 0·24)	0·55	−0·05 (−0·26 to 0·16)	0·63
Weight-for-length *Z* score	1642	−0·048 (−0·20 to 0·10)	0·53	−0·13 (−0·30 to 0·032)	0·11
**Household level**					
Thermotolerant coliforms per 100 mL water (colony-forming units)	1082	23·47 (−18·19 to 65·14)	0·27	11·93 (−30·51 to 54·38)	0·58
Drinking water obtained from an improved source§	7917	−0·057 (−0·16 to 0·046)	0·28	0·028 (−0·066 to 0·12)	0·56
Treatment of drinking water is adequate	7908	0·048 (−0·0086 to 0·11)	0·10	0·086 (0·029 to 0·14)	0·003
Improved sanitation facility¶	7917	0·0054 (0·054 to 0·065)	0·86	0·085 (0·015 to 0·16)	0·017
Structurally complete sanitation facility (ie, floor, walls, and roof)	7675	−0·0046 (−0·060 to 0·051)	0·87	0·065 (0·0013 to 0·13)	0·046
Faeces visible in courtyard	7916	0·014 (−0·0080 to 0·036)	0·21	0·00077 (−0·020 to 0·021)	0·94
Handwashing station observed, with soap and water	7916	−0·0049 (−0·020 to 0·011)	0·53	−0·0021 (−0·016 to 0·012)	0·77
Sanitary disposal of child faeces (for children <3 years)	5142	0·0094 (−0·036 to 0·055)	0·69	−0·012 (−0·056 to 0·033)	0·61
Household hunger (food security)	7920	0·95 (0·75 to 1·22)	0·70	1·15 (0·88 to 1·49)	0·31
**Child level**					
Exclusive breastfeeding (for infants <6 months)	896	−0·0027 (−0·074 to 0·069)	0·94	−0·00047 (−0·081 to 0·080)	0·99
Minimum dietary diversity (for children aged 6–23 months)	2683	0·024 (−0·032 to 0·080)	0·40	0·025 (−0·035 to 0·085)	0·41

Estimates for diarrhoea are prevalence ratios. Estimates for height-for-age, length-for-age, weight-for-height, or weight-for-length *Z* scores and thermotolerant coliforms per 100 mL water are β coefficients. Estimates for all intermediate outcomes except household hunger are risk differences. Estimates for household hunger are odds ratios.

* Total number of children or households.

† Adjusted for height-for-age *Z* score at baseline.

‡ Adjusted for weight-for-height *Z* score at baseline.

§ As defined by WHO and UNICEF, improved drinking water sources include piped water, public taps, tubewells, protected dug wells or springs, and rainwater.

¶ As defined by WHO and UNICEF, improved sanitation includes flush toilet, ventilated improved pit latrine, pit latrine with slab, and composting toilet.

Compared with control, the Classic intervention had a positive effect on reported household water treatment (RD 0·086, 95% CI 0·029–0·14; p=0·003), presence of improved sanitation facilities (0·085, 0·015–0·16; p=0·017), and presence of structurally complete sanitation facility (0·065, 0·0013–0·13; p=0·046). No effect of the Classic intervention was recorded on the remaining intermediate outcomes, including improved source of drinking water; sanitary disposal of children's faeces; presence of faeces in the courtyard; presence of a handwashing station with soap; exclusive breastfeeding for infants younger than 6 months; dietary diversity for infants aged 6–23 months; or household food security. Further analysis of the relation between adequate water treatment and the microbiological indicator of water quality showed no association (β −19·3, 95% CI −51·0 to 12·4; data not shown in table). In the Lite intervention, no effect was recorded on any intermediate outcomes versus control.

Among those assigned the Classic intervention, respondents or other household members reported attending a mean of 9·2 (SD 8·1) community health club sessions. Table 4 shows descriptive statistics and table 5 shows results of the per-protocol analysis of effects on primary, secondary, and intermediate outcomes for those allocated the Classic intervention who reported attending at least one session or all 20 sessions, compared with those allocated control. For those who reported attending at least one session, findings of the per-protocol analysis suggested a negative effect on weight-for-length *Z* score among children younger than 1 year (β coefficient −0·18, 95% CI −0·36 to −0·0058; p=0·04) and positive effects on reported household water treatment (RD 0·12, 95% CI 0·061–0·18; p<0·0001), presence of improved sanitation facility (0·089, 0·021–0·16; p=0·01), and presence of a structurally complete sanitation facility (0·062, 0·0057–0·12; p=0·03). For those who reported attending all 20 sessions, the per-protocol analysis suggested positive effects on reported household water treatment (RD 0·20, 95% CI 0·12–0·28; p<0·0001), presence of improved sanitation facility (0·14, 0·053–0·22; p=0·001), and presence of structurally complete sanitation facility (0·075, 0·0014–0·15; p=0·046). No other differences were noted.

**Table 4:** Descriptive statistics for per-protocol analysis of primary, secondary, and intermediate outcomes

		**Control**	**Attended at least one session (Classic)**	**Attended all 20 sessions (Classic)**
**Children measured at baseline and endline**				
Height-for-age *Z* score		−1·89 (1·06)	−1·91 (1·10)	−1·97 (0·97)
	Number of children	1378	840	160
Weight-for-height *Z* score		0·067 (0·92)	0·059 (0·97)	0·079 (0·95)
	Number of children	1377	843	163
**Children younger than 5 years**				
Diarrhoea		514/3617 (14%)	319/2247 (14%)	58/427 (14%)
Height-for-age *Z* score		−1·74 (1·18)	−1·80 (1·24)	−1·87 (1·13)
	Number of children	3320	2068	389
Weight-for-height *Z* score		0·077 (0·98)	0·031 (1·01)	0·054 (0·98)
	Number of children	3284	2034	384
**Children younger than 2 years**				
Diarrhoea		232/1210 (19%)	159/770 (21%)	30/139 (22%)
Length-for-age *Z* score		−1·54 (1·25)	−1·61 (1·34)	−1·72 (1·16)
	Number of children	1095	711	126
Weight-for-length *Z* score		0·18 (1·11)	0·071 (1·10)	0·077 (1·13)
	Number of children	1065	680	122
**Children younger than 1 year**				
Diarrhoea		109/644 (17%)	65/379 (17%)	14/76 (18%)
Length-for-age *Z* score		−1·21 (1·29)	−1·26 (1·39)	−1·36 (1·19)
	Number of children	602	359	70
Weight-for-length *Z* score		0·31 (1·17)	0·11 (1·22)	0·15 (1·20)
	Number of children	598	358	70
**Household level**				
Thermotolerant coliforms per 100 mL water (colony-forming units)		139·8 (230·9)	150·4 (239·1)	157·6 (245·9)
	Number of households*	362	238	54
Drinking water obtained from an improved source†		2134/2723 (78%)	1422/1751 (81%)	283/341 (83%)
Treatment of drinking water is adequate		1101/2720 (41%)	925/1751 (53%)	212/341 (62%)
Improved sanitation facility‡		805/2723 (30%)	652/1751 (37%)	149/341 (44%)
Structurally complete sanitation facility (ie, floor, walls, and roof)		695/2638 (26%)	538/1688 (32%)	115/333 (35%)
Handwashing station observed, with soap and water		47/2723 (2%)	27/1751 (2%)	13/341 (4%)
Sanitary disposal of child faeces (for children <3 years)		1115/1818 (61%)	698/1128 (62%)	136/205 (66%)

Data are number of children or number of households (%), or mean (SD).

* Water sampling was done in 10% of all study households.

† As defined by WHO and UNICEF, improved drinking water sources include piped water, public taps, tubewells, protected dug wells or springs, and rainwater.

‡ As defined by WHO and UNICEF, improved sanitation includes flush toilet, ventilated improved pit latrine, pit latrine with slab, and composting toilet.

**Table 5:** Per-protocol analysis of primary, secondary, and intermediate outcomes

	**Attended at least one session *vs* control**		**Attended all 20 sessions *vs* control**	
	Estimate (95% CI)	p	Estimate (95% CI)	p
**Children measured at baseline and endline**				
Height-for-age *Z* score*	−0·020 (−0·14 to 0·10)	0·76	−0·00022 (−0·19 to 0·19)	>0·99
Weight-for-height *Z* score†	−0·010 (−0·097 to 0·077)	0·82	−0·041 (−0·18 to 0·093)	0·55
**Children younger than 5 years**				
Diarrhoea	0·99 (0·85 to 1·16)	0·93	0·96 (0·77 to 1·20)	0·75
Height-for-age *Z* score	−0·050 (−0·19 to 0·093)	0·50	−0·13 (−0·31 to 0·039)	0·13
Weight-for-height *Z* score	−0·034 (−0·12 to 0·055)	0·45	−0·024 (−0·17 to 0·12)	0·74
**Children younger than 2 years**				
Diarrhoea	1·08 (0·87 to 1·34)	0·50	1·15 (0·78 to 1·68)	0·49
Length-for-age *Z* score	−0·073 (−0·25 to 0·10)	0·41	−0·18 (−0·42 to 0·056)	0·13
Weight-for-length *Z* score	−0·093 (−0·23 to 0·042)	0·18	−0·13 (−0·39 to 0·13)	0·34
**Children younger than 1 year**				
Diarrhoea	1·00 (0·73 to 1·39)	0·98	1·11 (0·65 to 1·88)	0·70
Length-for-age *Z* score	−0·08 (−0·31 to 0·15)	0·51	−0·15 (−0·50 to 0·19)	0·38
Weight-for-length *Z* score	−0·18 (−0·36 to −0·0058)	0·04	−0·17 (−0·51 to 0·18)	0·34
**Household level**				
Thermotolerant coliforms per 100 mL water (colony-forming units)	6·99 (−40·57 to 54·54)	0·77	21·70 (−48·72 to 92·12)	0·55
Drinking water obtained from an improved source‡§	0·043 (−0·026 to 0·11)	0·22	0·054 (−0·018 to 0·13)	0·14
Treatment of drinking water is adequate‡	0·12 (0·061 to 0·18)	<0·0001	0·20 (0·12 to 0·28)	<0·0001
Improved sanitation facility‡¶	0·089 (0·021 to 0·16)	0·01	0·14 (0·053 to 0·22)	0·001
Structurally complete sanitation facility (ie, floor, walls, and roof)‡	0·062 (0·0057 to 0·12)	0·03	0·075 (0·0014 to 0·15)	0·046
Handwashing station observed, with soap and water‡	−0·0005 (−0·014 to 0·013)	0·94	0·013 (−0·012 to 0·039)	0·30
Sanitary disposal of child faeces (for children <3 years)‡	0·004 (−0·042 to 0·051)	0·85	0·040 (−0·026 to 0·11)	0·24

Estimates for diarrhoea are prevalence ratios. Estimates for height-for-age, length-for-age, weight-for-height, or weight-for-length *Z* scores and thermotolerant coliforms per 100 mL water are β coefficients. Estimates for all intermediate outcomes are risk differences.

* Adjusted for baseline height-for-age *Z* score.

† Adjusted for baseline weight-for-height *Z* score.

‡ Adjusted for baseline values.

§ As defined by WHO and UNICEF, improved drinking water sources include piped water, public taps, tubewells, protected dug wells or springs, and rainwater.

¶ As defined by WHO and UNICEF, improved sanitation includes flush toilet, ventilated improved pit latrine, pit latrine with slab, and composting toilet.

## Discussion

The findings of our cluster-randomised trial indicate that community health clubs, as implemented under Rwanda's national CBEHPP campaign in this setting in western Rwanda, had no effect on health outcomes (diarrhoea and anthropometric measures) or faecal contamination of household drinking water. Our results suggest positive effects on several intermediate outcomes, including reported household water treatment and type and structure of sanitation facilities. A somewhat higher proportion of households in villages assigned the Classic intervention reported treating their drinking water, and were seen to have improved and completed the structure of sanitation facilities, compared with households in villages assigned the control. However, these augmented intermediate outcomes did not lead to decreases in caregiver-reported prevalence of diarrhoea or improvements in children's nutritional status.

The increased prevalence of reported household water treatment did not translate into a difference in water quality, as measured by the number of thermotolerant coliforms per 100 mL water. The reasons for this discrepancy are not clear, but could be attributable to social desirability bias in self-reported treatment of drinking water. Respondents in intervention villages might have learned that they should be using adequate methods but were not actually using those methods. This theory is supported by data showing no association between self-reported household water treatment and water quality. The results suggest a need for studies to use quantitative measurement of water quality to validate self-reports of household water treatment.

Per-protocol analysis of data from villages allocated the Classic intervention confirmed no effect on health outcomes or household water quality with self-reported attendance at all 20 sessions. Among intermediate outcomes, results suggested a dose-response relation with reported household water treatment and type and structure of sanitation facilities, but no effect on other key behaviours such as handwashing with soap and water. Results from any per-protocol analysis should be viewed with caution because of the risk of bias.^22^ People who were committed enough to attend community health club sessions might have been more likely to adhere to the target practices, irrespective of the intervention. Nevertheless, we judge that the per-protocol analysis confirmed the intention-to-treat analysis at the household level.

The absence of effect on height-for-age and weight-for-height *Z* scores might be attributable to the fact that the WASH components (the main targets of this intervention) are just one of many factors that might affect the nutritional status of a young child. The mean weight-for-height *Z* score for children younger than 5 years in this population is 0·07 (0·99), which means that children are in line with the global reference population, with limited scope for measurable improvements. With respect to determinants of child nutrition, the prevalence of exclusive breastfeeding for infants younger than 6 months in Rwanda is the highest in the world,^23^ and achieving additional substantial effects in this context would be difficult. Minimum dietary diversity among children aged 6–23 months, on the other hand, is low in this population, but we note that the community health club curriculum related to nutrition and diet is not fully aligned with global infant and young child feeding guidelines,^24^, ^25^ hence an absence of effect might not be surprising.

Our results point to several characteristics of this population that merit further attention. First, diarrhoea prevalence remains fairly high among children younger than 5 years. Second, the high prevalence of stunting alongside the low prevalence of wasting suggests that chronic undernutrition, but not acute malnutrition, is widespread among children younger than 5 years. The absence of acute malnutrition in a context of relatively high food insecurity, as measured by the household hunger scale, is surprising. Our results suggest that families could be accessing and providing their children with adequate calories but inadequate dietary diversity. Since both disease and inadequate dietary intake are immediate causes of undernutrition, programmes that reduce diarrhoea prevalence and increase dietary diversity could also reduce stunting.^26^ Finally, coverage of improved sanitation remains low, and handwashing stations remain almost non-existent among our study households.

Very little evidence exists on the effectiveness of the community health club or other community group-based models for health outcomes. In a previous study, the researchers concluded that the classic community health club intervention approach had potential to increase demand for sanitation and change related behaviours, but that study did not have a rigorous design that would have allowed for attribution to the intervention.^11^ Our results were similar with respect to sanitation but differed with respect to other behaviours, such as handwashing, and we saw no effect on diarrhoea or anthropometric measures. Few other studies have examined the effectiveness of community-group-based models for health outcomes, with the exception of women's groups for maternal and neonatal survival.^27^

Our results suggest that the community health club approach alone could be insufficient and that alternative or supplemental approaches might be needed to reduce the prevalence of diarrhoea in this population. For example, programmes could consider distributing water disinfection or filtration products to improve water quality.^28^, ^29^ Innovative approaches are needed to promote the presence of improved sanitation facilities and handwashing with soap, both of which are associated inversely with diarrhoea prevalence.^29^, ^30^, ^31^, ^32^ Alternatively, an integrated agriculture and nutrition approach has shown success in reducing the prevalence of diarrhoea in young children in Burkina Faso; further research is needed on the effectiveness of this intervention in other settings.^7^ In general, future programmes should promote intervention adherence and should actively target households for which the need is greatest.^33^

A key limitation of this study is the reliance on self-reported data for several variables, including diarrhoea, treatment of drinking water, disposal of child faeces, infant and young child feeding practices, and attendance at community health club sessions. Limitations of the per-protocol analysis include that it is prone to bias and that compliance is not easily defined. The per-protocol analysis could be particularly at risk of bias because of the use of self-reported data for attendance. We were able to reduce the risk of bias in the per-protocol analysis by adjusting for baseline differences, but results nevertheless need to be treated with caution. Finally, we did not assess the quality of delivery of the programme. Poor delivery could offer an explanation for the lack of an effect.^34^, ^35^, ^36^

The community health club model, as implemented under the CBEHPP in this setting in western Rwanda, had no effect on any main outcomes. Neither did it achieve the broader aims of the CBEHPP campaign, including zero open defecation and at least 80% hygienic latrine coverage. It had mixed results with respect to health behaviours, achieving positive results in some outcomes (eg, coverage of improved sanitation and type and structure of sanitation facility) but not in others (eg, handwashing with soap and water). Our results are strictly generalisable only to Rusizi district, but they are probably relevant to the rest of Rwanda; therefore, they raise questions about the value of implementing this intervention at scale with the goal of improving health outcomes.
